# New Dual Mode Gadolinium Nanoparticle Contrast Agent for Magnetic Resonance Imaging

**DOI:** 10.1371/journal.pone.0007628

**Published:** 2009-10-29

**Authors:** Ketan B. Ghaghada, Murali Ravoori, Divya Sabapathy, James Bankson, Vikas Kundra, Ananth Annapragada

**Affiliations:** 1 School of Health Information Sciences, The University of Texas Health Sciences Center at Houston, Houston, Texas, United States of America; 2 Department of Experimental Diagnostic Imaging, The University of Texas M.D. Anderson Cancer Center, Houston, Texas, United States of America; 3 Department of Imaging Physics, The University of Texas M.D. Anderson Cancer Center, Houston, Texas, United States of America; 4 Department of Diagnostic Radiology, The University of Texas M.D. Anderson Cancer Center, Houston, Texas, United States of America; National Institute on Drug Abuse, National Institutes of Health, United States of America

## Abstract

**Background:**

Liposomal-based gadolinium (Gd) nanoparticles have elicited significant interest for use as blood pool and molecular magnetic resonance imaging (MRI) contrast agents. Previous generations of liposomal MR agents contained gadolinium-chelates either within the interior of liposomes (core-encapsulated gadolinium liposomes) or presented on the surface of liposomes (surface-conjugated gadolinium liposomes). We hypothesized that a liposomal agent that contained both core-encapsulated gadolinium and surface-conjugated gadolinium, defined herein as dual-mode gadolinium (Dual-Gd) liposomes, would result in a significant improvement in nanoparticle-based T1 relaxivity over the previous generations of liposomal agents. In this study, we have developed and tested, both *in vitro* and *in vivo*, such a dual-mode liposomal-based gadolinium contrast agent.

**Methodology/Principal Findings:**

Three types of liposomal agents were fabricated: core-encapsulated, surface-conjugated and dual-mode gadolinium liposomes. *In vitro* physico-chemical characterizations of the agents were performed to determine particle size and elemental composition. Gadolinium-based and nanoparticle-based T1 relaxivities of various agents were determined in bovine plasma. Subsequently, the agents were tested *in vivo* for contrast-enhanced magnetic resonance angiography (CE-MRA) studies. Characterization of the agents demonstrated the highest gadolinium atoms per nanoparticle for Dual-Gd liposomes. *In vitro*, surface-conjugated gadolinium liposomes demonstrated the highest T1 relaxivity on a gadolinium-basis. However, Dual-Gd liposomes demonstrated the highest T1 relaxivity on a nanoparticle-basis. *In vivo*, Dual-Gd liposomes resulted in the highest signal-to-noise ratio (SNR) and contrast-to-noise ratio in CE-MRA studies.

**Conclusions/Significance:**

The dual-mode gadolinium liposomal contrast agent demonstrated higher particle-based T1 relaxivity, both *in vitro* and *in vivo*, compared to either the core-encapsulated or the surface-conjugated liposomal agent. The dual-mode gadolinium liposomes could enable reduced particle dose for use in CE-MRA and increased contrast sensitivity for use in molecular imaging.

## Introduction

T1-based MR contrast agents, exemplified by conventional low molecular-weight Gd-chelates have enabled contrast enhanced MRI for various applications such as tumor detection and characterization as well as vascular imaging [1]–[3]. For both of these applications, the T1 relaxivity of the agent can affect detection and anatomic demarcation of normal anatomy as well as pathology by reducing the T1 relaxation rate of tissue and generating positive contrast in T1-weighted images. In contrast-enhanced MR angiography (CE-MRA), conventional low molecular weight contrast agents extravasate into the extravascular-extracellular compartment (EEC) rather quickly, leaving a short window for imaging the vessel lumen. This can lead to blurring of the resulting images and low vessel conspicuity, particularly for smaller vessels. The problem is further exacerbated if the bolus is not appropriately timed, which can severely limit imaging of both large and small vessels. One approach to circumventing the need for accurate bolus timing involves the use of agents with longer intravascular half-life [4], [5]. Slower extravasation reduces blurring at vessel boundaries and increases the contrast-to-noise (CNR) ratio between vascular structures and surrounding tissues. In addition, an agent with high T1 relaxivity can also improve imaging of small features by increasing the amount of signal generated within the vascular space in CE-MRA [6], [7].

The T1 relaxivity and *in vivo* half-life of a contrast agent becomes even more critical in small animal imaging, where the vessels are much smaller than in humans, resulting in longer scan times and therefore increased propensity to extravasate into the extravascular space. In recent work, this shortcoming was highlighted in small animal imaging [8], where even under optimized imaging conditions in a 7T scanner, it was not possible to clearly image the intercostal and spinal vasculature in a rat with conventional low molecular-weight Gd-chelates. Thus, agents that remain intravascular and have greater T1-relaxivity per unit of contrast agent are needed in order to amplify signal, improve CNR, and enable vascular imaging with high vessel conspicuity.

The development of such agents is also being pursued in the field of molecular MRI where the targets of interest, such as tumor cells or cell-associated molecules, are present at micro- or nano- molar concentrations. One approach to prepare such an agent is to associate Gd-chelates with a nanoparticle. Several nanoparticle-based platforms have been utilized to develop signal amplification contrast agents [9], [10], [11]. One class of nanoparticles, liposomes, has provided a unique platform for development of MR contrast agents. Liposomes have been used for preparation of two separate Gd-based constructs: core-encapsulated Gd (CE-Gd) liposomes wherein the Gd-chelates are encapsulated in the interior core of liposomes [12], [13]; and surface-conjugated Gd (SC-Gd) liposomes wherein the Gd-chelates are presented on the surface of liposomes [14]. Compared to conventional Gd-chelates, liposomal-Gd agents have long *in vivo* half life and have very low propensity to extravasate except at regions of ‘leaky’ vasculature, such as tumor blood vessels [15], [16], [17]. As a result, these agents provide an extended imaging window for acquisition of high-resolution images, thus enabling excellent small vessel depiction. This has been demonstrated previously by imaging of sub-millimeter vascular features of the CNS neurovasculature [8] and the cardiovascular system [18] in small animals. From a molecular MRI perspective, such nanoparticle agents are able to deliver a high payload of Gd-chelates to the target site and therefore amplify signal. In addition to molecular targeting, such nanoparticle agents would also find applications in highlighting low levels of vascular leak as observed in tumors [19].

**Figure 1:**
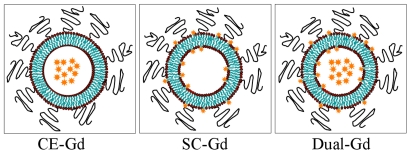
Schematic of various liposomal-Gd agents. Core-encapsulated gadolinium (CE-Gd) liposomes contain conventional low molecular-weight Gd-chelates in the core interior of the liposomes, surface-conjugate gadolinium (SC-Gd) liposomes contain Gd-chelates conjugated on the internal and external surface of the liposome bilayer, Dual-Gd liposomes contain both core-encapsulated and surface-conjugated Gd-chelates. The stars represent Gd-chelates.

We reasoned that the full capability of liposomes to amplify signal has not been exploited and therefore tested whether liposomes that *both encapsulate* and *display gadolinium* on their *surface* would exhibit increased T1 relaxivity *in vitro* and whether this leads to further signal enhancement *in vivo*. In this work, we therefore investigated such “Dual-Gd” agents. In the rest of this paper, we use the term CE-Gd (Core-Encapsulated) for nanoparticles that encapsulated gadolinium within the core-interior, SC-Gd (Surface-Conjugated) for those nanoparticles that have gadolinium conjugated on the surface, and Dual-Gd for particles that have both core-encapsulated and surface-conjugated gadolinium (Figure 1).

## Materials and Methods

### 1.1. Ethics Statement

All animals were handled in accordance with good animal practice as defined by the relevant national and/or local animal welfare bodies, and all animal work was approved by the Institutional Animal Care and Use Committee (IACUC) of MD Anderson Cancer Center. The MD Anderson Cancer Center animal management program is accredited by the American Association for the Accreditation of Laboratory Animal Care and meets National Institute of Health standards as set forth in the “Guide for the Care and Use of Laboratory Animals” (DHHS Publication No. (NIH) 85–23, Revised 1985). The institution also accepts as mandatory the PHS “Policy on Humane Care and Use of Laboratory Animals by Awardee Institutions” and “NIH Principles for the Utilization and Care of Vertebrate Animals Used in Testing, Research and Training”.

### 1.2. Preparation of liposomal contrast agents

For preparation of CE-Gd liposomes, a lipid mixture consisting of 1,2-Dipalmitoyl-*sn*-glycero-3-phosphocholine (DPPC) (Genzyme, MA), Cholesterol (Sigma-Aldrich, St Louis, MO) and 1,2-distearoyl-*sn*-glycero-3-phosphoethanolamine-N-[methoxy(poly(ethylene glycol))-2000] (mPEG2000-DSPE) (Genzyme, MA) in the molar ratio 55∶40∶5 was dissolved in ethanol. Subsequently, the ethanol solution was mixed with a solution of gadobenate dimeglumine (Multihance^®^, Gd-BOPTA, 500 mM Gd) to achieve a lipid concentration of 150 mM. The solution was stirred for 90 minutes at 60°C and then sequentially extruded on a Lipex Thermoline extruder (Northern Lipids, Vancouver, British Columbia, Canada) with three passes through a 400 nm Nuclepore membrane (Waterman, Newton, MA), five passes through a 200 nm and ten passes through a 100 nm membrane. The resulting solution was diafiltered using a MicroKros^®^ module (Spectrum Laboratories, CA) of 500 kDa molecular weight cut-off to remove unencapsulated Gd-chelate molecules.

For SC-Gd liposomes preparation, a lipid mixture consisting of DPPC, Gd-DTPA bis(stearylamide) (Gd-DTPA-BSA) (IQsynthesis, St Louis, MO), Cholesterol and mPEG2000-DSPE in the molar ratio 30∶25∶40∶5 was dissolved in a chloroform: methanol (1∶1 v/v) mixture. The solvent mixture was evaporated to dryness under vacuum and the lipid contents were hydrated with 150 mM saline to achieve a lipid concentration of 40 mM. The solution was stirred for 90 minutes at 60°C and then sequentially extruded with five passes through a 400 nm Nuclepore membrane, seven passes through a 200 nm Nuclepore membrane and ten passes through a 100 nm Nuclepore membrane.

For preparation of Dual-Gd liposomes, a lipid mixture consisting of DPPC, Gd-DTPA-BSA, Cholesterol and mPEG2000-DSPE in the molar ratio 30∶25∶40∶5 was dissolved in a chloroform: methanol (1∶1 v/v) mixture. The solvent mixture was evaporated to dryness under vacuum and the lipid contents were hydrated with a solution of gadobenate dimeglumine (Multihance^®^, Gd-BOPTA, 500 mM Gd) to achieve a lipid concentration of 40 mM. The solution was stirred for 90 minutes at 60°C and then sequentially extruded with five passes through a 400 nm Nuclepore membrane, seven passes through a 200 nm Nuclepore membrane and ten passes through a 100 nm Nuclepore membrane. The resulting solution was diafiltered using a MicroKros^®^ module (Spectrum Laboratories, CA) of 500 kDa molecular weight cut-off to remove unencapsulated Gd-chelate molecules. To demonstrate reproducibility in the synthesis process, two different batches of each liposomal agent were prepared and characterized as described below.

### 1.3. Characterization of liposomal agents

#### 1.3.1. Particle size and composition

The size distribution of liposomes in the final formulation was determined by dynamic light scattering (DLS) using a ZetaPlus Analyzer (Brookhaven Instruments, Chapel House, UK) at 25°C. The gadolinium and phosphorus content of liposomal formulations were quantified using inductively coupled plasma optical emission spectroscopy (ICPOES; Model Optima 4300D, Perkin Elmer, Norwalk, CT) operating at a wavelength of 336.223 nm for gadolinium and 213.617 nm for phosphorus. The number of gadolinium atoms per liposome was calculated based on the Gd:P ratio, mean liposome diameter, and the respective phospholipid molar composition for each formulation.

#### 1.3.2. Measurement of Gd-based molar T1 relaxivity

Samples with gadolinium concentrations ranging between 0.25 mM–2 mM (5 samples) were prepared by diluting the liposomal solutions in bovine plasma. T1 relaxation measurements were performed on a 60 MHz Minispec MQ series benchtop relaxometer (Bruker Optics) at 37°C. The longitudinal relaxation rates (R1) of the diluted samples were obtained using an inversion recovery method. A plot of R1 versus gadolinium concentration yielded a straight line with the slope defined as the T1 relaxivity (r1). To demonstrate reproducibility in T1 relaxation times, measurements were repeated after one week by preparing fresh dilutions in bovine plasma.

#### 1.3.3. Measurement of T1 relaxation rates for different lipid dose

The T1 relaxation rates (R1) of three liposomal contrast agents were also compared on a particle basis. Dilutions were done on a lipid-dose basis i.e., amount of lipid administered (mg) per body weight (kg). Diluted samples were prepared in bovine plasma to achieve lipid concentrations ranging between 40–400 mg lipid/kg of human body weight. The chosen concentration range represents contrast agent doses that are likely to enable sufficient SNR for *in vivo* imaging. To demonstrate stability and reproducibility in T1 relaxation times, measurements were repeated after one week by preparing fresh dilutions in bovine plasma.

#### 1.3.4. Calculation of nanoparticle-based T1 relaxivity

To calculate T1 relaxivity on a nanoparticle-basis, the above lipid-dose was converted into nanoparticle concentration. An average lipid molecular weight was determined using the molecular weight and mole fraction of each lipid used in liposome preparation. The nanoparticle concentration was then determined using the number of lipids per liposome and average lipid molecular weight. A plot of R1 versus liposome concentration yielded a straight line with the slope defined as the nanoparticle-based T1 relaxivity (r1).

### 1.4. *In vivo* Imaging

#### 1.4.1. Animal preparation

Six nude mice were used for the studies. For *in vivo* comparison of different liposomal formulations, the agents were intravenously administered via the tail vein at a lipid dose of 200 mg/kg. The corresponding gadolinium doses were 0.07, 0.08 and 0.15 mmoles/kg for CE-Gd, SC-Gd and Dual-Gd, respectively. The same animals were used for all three agents, in a randomized order. On day 1, each mouse received a randomly selected agent (1: CE-Gd, 2: SC-Gd, 3: Dual-Gd). After imaging, the animals were returned to their cages for a minimum of 3 days. At the second imaging session, mice received the next agent in the list, i.e., mice that had received CE-Gd earlier then received SC-Gd, mice that had received SC-Gd earlier then received Dual-Gd and mice that had received Dual-Gd earlier then received CE-Gd. The animals then progressed to the next contrast agent in the third imaging session. Inhalation of 2% isofluorane was used for anesthesia.

#### 1.4.2. MR Imaging Protocol

All MR studies were performed on a 4.7T scanner (Bruker BioSpec, 47/40 USR, Bruker Biospin, Billerica, MA) using a 60-mm gradient insert and a volume resonator with a 35 mm inner diameter. Animals were anesthesized and placed head first and prone on a positioning sled. Orthogonal 3-plane scout scans were initially acquired for animal positioning. Pre-contrast and post-contrast MRA images were acquired using a heavily T1-weighted 3D fast spoiled gradient echo sequence (FSPGR). Scans were acquired with the following imaging parameters: repetition time (TR) = 5.0 ms; echo time (TE) = 2.1 ms; flip angle (FA) = 30°; field of view (FOV) = 30×30×30 mm^3^; Image matrix = 128×128×128; number of signal averages = 5. This resulted in an isotropic voxel size of 320 µm. The total scan time was under seven minutes. Maximum intensity projection (MIP) images were performed and analyzed.

### 1.5. Image Analysis

Signal to noise ratios (SNRs) and contrast-to-noise ratios (CNRs) were calculated for regions of interest in the jugular veins and muscle. The SNR was calculated as 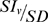 where, *SI_v_* is the mean signal intensity within the blood vessel and SD is the standard deviation of the signal intensity within the background (air). The CNR was defined as 
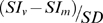
 where, *SI_m_* is the mean signal intensity in the muscle region. Pre-contrast SNRs were subtracted from post-contrast SNRs to minimize differences in baseline signals from animal to animal. Similarly, pre-contrast CNRs were subtracted from post-contrast CNRs. SNRs and CNRs calculations were performed for each animal and then average values were reported for the group (n = 6).

### 1.6. Statistical Analysis

For comparing groups, two-tailed t-tests were performed using spreadsheet software (Microsoft Office Excel 2003, Microsoft, Seatle, WA). A P-value of less than 0.05 was considered statistically significant.

## Results

The three liposomal-gadolinium formulations demonstrated good reproducibility in the fabrication process (Table 1). Size analysis of liposomes indicated particles of approximately 100 nm in diameter. The low polydispersity index for various formulations indicated narrow size distributions. More than 95% of liposomes in all the three formulations were below 150 nm. No significant changes in size distribution were observed over a one month period (data not shown). The Dual-Gd liposomes had the highest gadolinium to phospholipid (Gd:P) ratio. The calculated number of Gd per liposome were also highest for the Dual-Gd formulation. This was due to the presence of two gadolinium pools in Dual-Gd liposomes – the core-encapsulated pool and the surface-conjugated pool. Since the lipid composition and molarity for SC-Gd liposomes is similar to Dual-Gd liposomes, one would expect similar surface-Gd per liposome for both agents. The higher Gd per liposome observed for Dual-Gd compared to the sum total of CE-Gd and SC-Gd liposomes is therefore most likely a result of increased encapsulated Gd fraction compared to the CE-Gd liposomes. This is most likely due to the larger liposome size obtained for Dual-Gd liposomes compared to the CE-Gd liposomes.

**Table 1:** Characterization of liposomal-Gd formulations.

Agent		Mean Diameter (nm)	Polydispersity Index	Cumulative particle size distribution			Gd:P ratio	Gd per liposome
				% <200 nm	% <150 nm	% <100 nm		
CE-Gd	Batch-1	99±2	0.054	100	≥97	≥75	0.398	34783
	Batch-2	94±2	0.022	100	≥99	≥80	0.375	29420
SC-Gd	Batch-1	110±3	0.012	100	≥95	≥60	0.784	49743
	Batch-2	109±3	0.035	100	≥96	≥60	0.782	48685
Dual-Gd	Batch-1	114±4	0.042	100	≥93	≥53	1.648	112590
	Batch-2	107±3	0.054	100	≥97	≥62	1.560	93462

**Figure 2:**
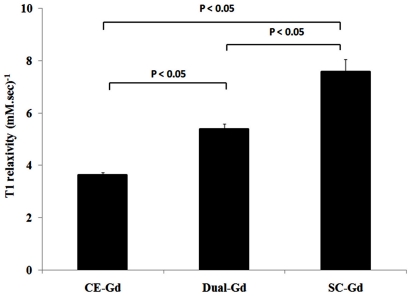
T1 relaxivity of liposomal-Gd formulations on a per Gadolinium basis. Measurements were performed at 1.5 Tesla MR field strength in bovine plasma at 37°C. Each value is significantly different (p<0.05).

The Gd-based molar T1 relaxivities (r1) of the three liposomal formulations were also compared. SC-Gd liposomes demonstrated the highest r1 whereas CE-Gd liposomes had the lowest (Figure 2). The r1 of Dual-Gd was significantly lower than that of SC-Gd (p<0.05). The higher r1 observed for SC-Gd is a result of more liposomal particles present per unit concentration of Gd. Consequently, more Gd atoms are exposed to the bulk water molecules which eventually results in an enhanced T1 relaxation effect. No significant changes in the r1 were observed over a one week period (data not shown).

**Figure 3:**
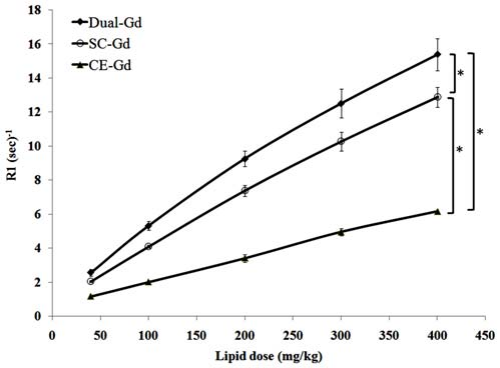
T1 relaxation rates (R1) of liposomal-Gd formulations for different lipid doses. Measurements were performed at 1.5 Tesla MR field strength in bovine plasma at 37°C. For each lipid dose, the R1 values were significantly different for each of the liposomal-Gd agent (* corresponds to p<0.05).

**Figure 4:**
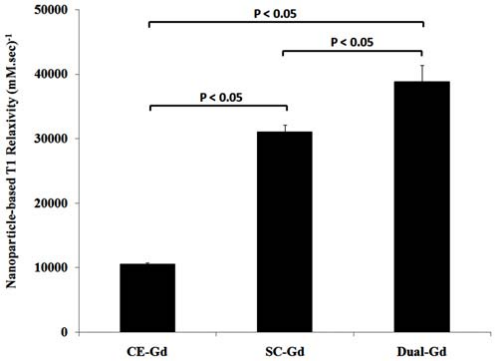
T1 relaxivity of liposomal-Gd formulations on a per nanoparticle basis.

To compare the relaxation properties of liposomal formulations on a particle basis, *in vitro* T1 relaxation rates (R1) were measured for diluted samples prepared in bovine plasma. The dilutions were performed on a lipid-dose basis i.e., amount of lipid administered (mg) per unit of body weight (kg). The Dual-Gd and SC-Gd liposomes had at least two-fold higher relaxation rates compared to CE-Gd liposomes at all lipid doses (p<0.05, Figure 3). The Dual-Gd liposomes demonstrated the highest R1 on a particle-basis among the three formulations (p<0.05 Dual-Gd vs SC-Gd, Figure 2). The higher R1 for Dual-Gd liposomes is a combined effect of surface-conjugated and core-encapsulated gadolinium which causes more protons to relax compared to either SC-Gd or CE-Gd liposomes. This is also evident in the T1 relaxivities calculated on a nanoparticle-basis (Figure 4). Dual-Gd liposomes demonstrated the highest nanoparticle-based T1 relaxivity. The nanoparticle-based T1 relaxivities were more than three orders of magnitude (2000–8000) higher than conventional low molecular-weight contrast agents such as Gd-DTPA. The high T1 relaxivities is a result of their ability to carry several thousands of Gd-chelates per nanoparticle.

**Figure 5:**
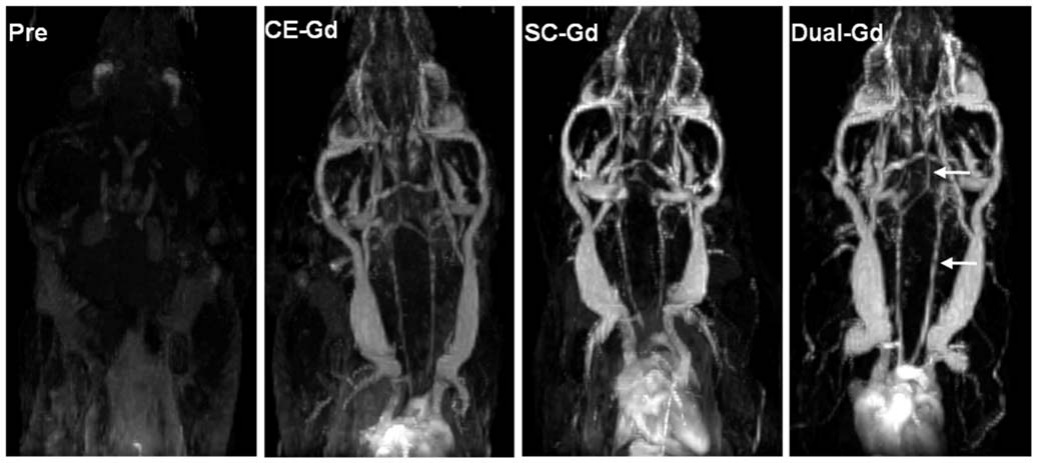
*In vivo* comparison of liposomal-Gd contrast agents. Coronal maximum intensity projection (MIP) images of the head and thorax in mice acquired pre-contrast, post CE-Gd liposomes, post SC-Gd liposomes and post Dual-Gd liposomes. The contrast agents were administered intravenously at a lipid dose of 200 mg/kg. Please note the increased signal in the vessels compared to background and the high vessel conspicuity for smaller vessels (arrows in the Dual-Gd image). All images were acquired in different animals using the 3D-FSPGR sequence. The MIP images are presented at identical gray-scale levels.

**Figure 6:**
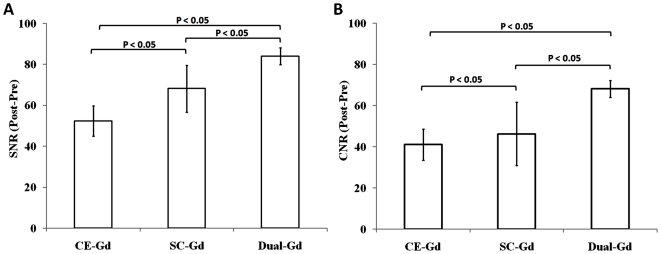
*In vivo* comparison of signal to noise ratios (SNR) (Fig. A) and contrast to noise ratios (CNR) (Fig. B) for different liposomal-Gd agents. Each agent was administered in mice at a lipid dose of 200 mg/kg. There was significant difference within the SNRs and CNRs of various contrast agents (p<0.05).

To compare different liposomal formulations *in vivo*, the agents were administered at identical lipid dose i.e., resulting in equivalent liposome concentration in the blood. For comparison purposes, all maximum intensity projection (MIP) images were processed to identical brightness/contrast levels. All three agents demonstrated visualization of large vessels (Figure 5). However, Dual-Gd and SC-Gd liposomes better demonstrated small vessel features as seen in the coronal MIP images. Increased signal and relatively lower background were noted in the Dual-Gd images, in addition, smaller vessels became even more conspicuous. This is reflected in the SNR and CNR values (Figure 6), which show that Dual-Gd had the highest SNR and CNR, followed by SC-Gd, and then CE-Gd. The differences in SNR and CNR values for all three agents were statistically significant (p<0.05). The better demonstration of vascular features by Dual-Gd, relative to the other two agents, is a result of higher Gd delivered (both surface-conjugated and core-encapsulated) per nanoparticle.

## Discussion

The need to increase MRI image contrast, both as measured by signal-to-noise (overall brightness) and contrast-to-noise (feature conspicuity) ratio is a constant thrust in the field of contrast agent development. Nanoparticle-based contrast agents are particularly interesting because they can be used to amplify signal by delivering more contrast agent molecules to an area of interest; therefore, improving both overall signal and feature conspicuity. For vascular imaging, SNR and CNR may be improved by nanoparticles contrast agents with (1) decreased propensity for indiscriminate extravasation, thus, reducing vessel blurring; and, (2) long intravascular circulation time, thus, reducing the need for bolus tracking. This permits acquisition of high-resolution scans with several image signal averages. This should aid small feature analysis that is critical in imaging of small animals and in clinical decision making.

In addition, the capability to deliver a large payload of Gd is also quite attractive for molecular imaging. Molecular targeting of individual contrast molecules results in a typical 1∶1 target-to-readout ratio. Since molecular targets, such as receptors present in the vasculature, are often present in micro- or nano-molar concentrations, readouts will also typically be in this concentration range. This has made molecular imaging the domain primarily of nuclear imaging methods, which have high sensitivity to detect such low concentration ranges. With nanoparticles, the ratio of contrast agent to target can be dramatically increased so that thousands of imaging agents are localized per particle bound to the target. Thus, measuring the relaxivity on a particle-molar basis is a good way of estimating the signal achievable for nanoparticle-based contrast agents [20].

Several macromolecular and nanoparticle-based platforms have been investigated for the development of blood pool and molecular MRI contrast agents. MS-325, an albumin-binding Gd-chelate was recently approved for use as a blood pool MR contrast agent in USA. While the agent is known to demonstrate persistent blood pool contrast, the mechanism of action is based on transient interactions with human serum albumin (HSA) and therefore is likely to exhibit variability in signal enhancement due to on-off binding [21]. In the nanoparticle-domain, three major platforms have been investigated. Lipid-based paramagnetic perfluorocarbon (PFC) nanoparticles have been used as molecular MRI contrast agents for several targets [9]. The mean diameter of PFC-nanoparticles is around 250 nm, thus enabling higher surface incorporation of Gd per nanoparticle and therefore higher nanoparticle-based relaxivity compared to smaller Dual-Gd agents described here. Dendrimers-based gadolinium nanoparticles have also been extensively investigated for use as blood pool and molecular MRI contrast agents [11]. The use of dendrimer as a platform enables synthesis of a broad range of particle sizes, however, the payload of Gd that can be delivered is limited by the number of conjugation sites available on the dendrimer. Liposomes have also been extensively investigated for use as contrast agents. A particular problem with CE-Gd liposomes has been the inability to generate a large amount of T1 relaxivity due to limited proton exchange between the interior and exterior of liposomes because of the liposomal bilayer [13]. The presentation of Gd-chelates on the surface of nanoparticles in SC-Gd liposomes have resulted in equal or higher T1 relaxivities compared to conventional contrast agents. However, the number of sites for Gd-chelate conjugation on the liposome are limited to about 25% of the number of surface lipid molecules because larger percentages tend to destabilize the membrane [14]. Although this limits the total enhancement that is achievable using surface Gd, alternate liposome-based structures that exhibit higher relaxivity are feasible and could further improve image quality. Liposomes containing Gd-chelates complexed to very short polymeric chains of ethylene glycol (PEG) have also been investigated [22]. The complexation of Gd on the flexible polymers resulted in higher T1 relaxivity on a Gd-basis. However, the relatively short PEG chains (2 monomer units) used in their preparation would result in faster clearance of their agent.

The T1 relaxivities of free Gd-chelates are on the order of 3–5 (mM.sec)^−1^; whereas, Dual-Gd provides a nanoparticle-based T1 relaxivity of 35000 (mM.sec)^−1^, which is approximately 10^4^ times higher than that of free Gd-chelate. PFC-based nanoparticles have been reported to have higher nanoparticle-based relaxivities than Dual-Gd liposomal agents [20]. The high relaxivities of PFC nanoparticles is due to their broad and large particle size distribution and the fact that all Gd-chelates, present in the lipid monolayer, are exposed to the bulk water. The differences in mean particle size diameter and size distribution between Dual-Gd liposomes and PFC-based nanoparticles therefore precludes direct comparison of such agents on a nanoparticle-basis. However, the development of such nanoparticles with high signal amplification begins to bring molecular imaging into the realm of MRI. This in turn has huge potential advantages since MRI, unlike nuclear imaging techniques, can demonstrate anatomy along with the molecular target, with enormously high spatial resolution. Thus, Dual-Gd is a key step on this path.

Another key parameter that characterizes a nanoparticle-based contrast agent is the number of particles injected in order to generate sufficient image contrast. This has implications ranging from the potential for infusion-related reactions to the ultimate clearance route and toxicity. Infusion related reactions, which can cause complement-activation related pseudoallergies (CARPA), are sensitive to physicochemical properties of nanoparticles [23]. Clearance of nanoparticles is usually via the reticulo-endothelial system (RES), and a high particle load could lead to RES overload, thus compromising the body's ability to clear other particulate species. A lower dose of a contrast agent with high relaxivity can be injected to achieve the same signal enhancement as a larger quantity of an agent with lower relaxivity. Thus, the relaxivity on a molar basis of nanoparticles is critically important to avoid toxicity and other complications.

Dual-Gd, being based on the well known PEGylated (Stealth) liposome platform, has several advantages. First, it is a versatile platform, as demonstrated by the easy preparation of 3 different variants in this work (CE, SC and Dual). Second, there is much known about the safety and disposition of such liposomes in the body; indeed, there are therapeutic products already in clinical use that are based on this platform [24]. However, the safety and disposition of Gd-based liposomal agents needs to be further investigated. The long circulating property of Stealth liposomes, coupled with their controlled extravasation in regions of vascular compromise, make this class of agents quite attractive for visualizing vascular lesions. Moreover, there is much known about the molecular targeting of Stealth liposomes [25]–[27]. To capitalize on these advantages, improving the T1 relaxivity per particle is critical. The orders of magnitude improvement in relaxivity achieved per nanoparticle with Dual-Gd compared to conventional Gd-chelate agents should enable improved vascular imaging and paves the way for molecularly-targeted MR imaging.

### Conclusions

We have demonstrated a new nanoparticle-based Gd agent for T1-weighted MR imaging. The new agent, named Dual-Gd liposomal agent, combines features of two previously described agents, CE-Gd (core-encapsulated Gd liposome) and SC-Gd (surface-conjugated Gd liposome) to create a new entity that is capable of delivering a higher concentration of Gd, thus greater signal enhancement per particle. Upon *in vivo* imaging, the Dual-Gd resulted in both improved SNR and CNR. Among other applications, the agent should find use in vascular imaging, particularly for evaluation of small features that can be critical for clinical decision making and for imaging of small animals; as well as, in molecular imaging, where the number of imaging agents per particle bound to the target is critical for creating sufficient signal for target identification.
